# A Single Intravenous AAV9 Injection Mediates Bilateral Gene Transfer to the Adult Mouse Retina

**DOI:** 10.1371/journal.pone.0061618

**Published:** 2013-04-15

**Authors:** Alexis-Pierre Bemelmans, Sandra Duqué, Christel Rivière, Stéphanie Astord, Mélissa Desrosiers, Thibault Marais, José-Alain Sahel, Thomas Voit, Martine Barkats

**Affiliations:** 1 INSERM, U968, Paris, France; 2 Institut de la Vision, Université Pierre et Marie Curie Paris 6 - UM80, Paris, France; 3 UMR_7210, CNRS, Paris, France; 4 Molecular Imaging Research Center (MIRCen) and CNRS URA2210, Commissariat à l’Energie Atomique et aux Energies Alternatives (CEA), Département des Sciences du Vivant (DSV), Institut d’Imagerie Biomédicale (I2BM), Fontenay-aux-Roses, France; 5 UM76 UPMC-AIM UMR S974, INSERM U 974, CNRS UMR 7215, Institut de Myologie, Université Pierre et Marie Curie Paris 6, Paris, France; 6 Généthon, Evry, France; 7 INSERM-DHOS CIC 503, Centre Hospitalier National d’Ophtalmologie des Quinze-Vingts, Paris, France; University of Florida, United States of America

## Abstract

Widespread gene delivery to the retina is an important challenge for the treatment of retinal diseases, such as retinal dystrophies. We and others have recently shown that the intravenous injection of a self-complementary (sc) AAV9 vector can direct efficient cell transduction in the central nervous system, in both neonatal and adult animals. We show here that the intravenous injection of scAAV9 encoding green fluorescent protein (GFP) resulted in gene transfer to all layers of the retina in adult mice, despite the presence of a mature blood-eye barrier. Cell morphology studies and double-labeling with retinal cell-specific markers showed that GFP was expressed in retinal pigment epithelium cells, photoreceptors, bipolar cells, Müller cells and retinal ganglion cells. The cells on the inner side of the retina, including retinal ganglion cells in particular, were transduced with the highest efficiency. Quantification of the cell population co-expressing GFP and Brn-3a showed that 45% of the retinal ganglion cells were efficiently transduced after intravenous scAAV9-GFP injection in adult mice. This study provides the first demonstration that a single intravenous scAAV9 injection can deliver transgenes to the retinas of both eyes in adult mice, suggesting that this vector serotype is able to cross mature blood-eye barriers. This intravascular gene transfer approach, by eliminating the potential invasiveness of ocular surgery, could constitute an alternative when fragility of the retina precludes subretinal or intravitreal injections of viral vectors, opening up new possibilities for gene therapy for retinal diseases.

## Introduction

A number of inherited human blindness disorders, some monogenic and others with multi-factorial inheritance, are particularly amenable to gene therapy. It would therefore be theoretically possible to treat retinal diseases, such as retinitis pigmentosa (RP), which is caused by gene mutations in photoreceptors or retinal pigment epithelium (RPE) [1], and glaucoma, which is caused by gene mutations in retinal ganglion cells (RGC) [2], by transferring specific genes into the appropriate retinal cell population. The delivery of transgenes to retinal cells in animal models generally requires subretinal or intravitreal injections of viral vectors, such as adeno-associated virus (AAV) or lentiviral vectors. Genes can generally be delivered to the inner side of the retina through the intravitreal injection of AAV vectors but, due to vector accumulation at the vitreoretinal junction, only localized transgene expression can be obtained in RGC, and deep retinal layers have never been successfully transduced in healthy retina [3]–[5]. By contrast, the subretinal route of delivery can be used to target the RPE and/or the photoreceptors with lentiviral or AAV vectors [6]–[10]. AAV is a particularly promising vector for gene therapy for retinal diseases, due to its weak immunogenicity, its ability to infect all retinal cell types and its potential for the long-term expression of transgenes [3], [11]. Several clinical trials have recently shown that the subretinal injection of RPE65-encoding AAV yields significant visual improvement in patients with Leber’s congenital amaurosis (LCA), a severe form of retinal degeneration affecting children [12]–[14].

The subretinal injection of gene vectors is generally considered to be safe [6], [15], but this surgical procedure induces detachment of the retina at the site of injection, leading to localized trauma and possible retinal thinning and cell destruction [16]–[18]. If prolonged, retinal detachment can induce the apoptotic cell death of photoreceptor cells, leading to a loss of vision [19]. In the first clinical trials of gene therapy for LCA [12]–[14], the subretinal injection of RPE65-expressing AAV caused temporary retinal detachment, which resolved spontaneously in most cases. However, the development of a macular hole in one case highlights the risks of such surgery [20], [21]. Subretinal gene vector delivery is also of questionable value in gene therapy for retinal disorders increasing the likelihood of retinal detachment, such as X-linked juvenile retinoschisis [5], or diseases affecting the subretinal space, such as the wet-form of age-related macular degeneration, in which the development of subretinal choroidal neovascular membranes or subretinal hemorrhage is responsible for most of the vision loss [22]. Finally, subretinal injection usually limits transgene delivery to the area surrounding the injection site, and this is not ideal for the treatment of diseases requiring the transduction of cells throughout the retina [5]. The translation of retinal gene transfer into clinical practice might therefore require alternative delivery routes to subretinal injection.

The transfer of genes to cells throughout the retinas of both eyes *via* a single systemic injection of viral gene vectors would constitute an attractive non invasive strategy for the treatment of retinal diseases. However, systemic gene transfer to the retina is hampered by the tight junctions of the blood-eye barrier, which prevents the passage of viral vectors from the bloodstream into the subretinal space, particularly in adults [23]. We and others have shown that the self-complementary AAV9 vector (scAAV9) has a remarkable ability to mediate widespread transgene expression in the brain and spinal cord following its intravenous injection into both neonatal and adult animals [24]–[26], suggesting that this vector can cross the blood-brain barrier (BBB). The therapeutic potential of this systemic approach was recently demonstrated in a mouse model of spinal muscular atrophy (SMA), a devastating neuromuscular disorder caused by mutations or deletions of the “Survival of Motor Neuron” (*SMN*) gene [27]–[30]. In these pioneering studies, mice intravenously injected with *SMN*-encoding scAAV9 during the perinatal period displayed an impressive rescue of SMA. The basis of this robust transduction of central nervous system (CNS) cells after the systemic delivery of scAAV9 remains unclear. It is thought to involve differential BBB transport, but it remains unclear how AAV9 crosses the BBB and whether this mechanism is different from that of other serotypes *in vivo*. The superiority of scAAV9 for the systemic transduction of nerve cells may be due to various factors, including capsid-interacting blood factors, strong neural cell tropism or intracellular trafficking, the rapid uncoating of virion shells in cells and delayed blood clearance [31], [32].

In this study, we investigated whether the systemic injection of scAAV9 could mediate transduction of the retina in adult mice despite the presence of functional blood-eye barriers. We found that the intravenous injection of scAAV9 into adult mice resulted in gene transfer to all cell layers of the retina, with the predominant transduction of RGC and ciliary bodies. This study suggests that this vector could cross mature blood-eye barriers, and constitutes the basis for future development of a non invasive alternative to the current methods of viral gene delivery to the retina.

## Materials and Methods

### Animals

This study was performed on adult (6 to 8 weeks old, female) C57Bl6 mice purchased from Charles River Laboratories (Les Oncins, France). All animal experiments were carried out in accordance with European guidelines for the care and use of experimental animals.

### AAV Vector Production

AAV vectors express GFP or mouse secreted alkaline phosphatase (mSEAP) under the control of the cytomegalovirus immediate early (CMV) promoter. Self-complementary genome-containing plasmids were constructed by deleting the D sequence and the terminal resolution site from one of the inverted terminal repeats. The production of serotype 9 AAV has been described elsewhere [24]. Briefly, pseudotyped AAV9 and AAV2 vectors were generated by packaging AAV2-based recombinant single-stranded (ss) or self-complementary (sc) genomes into the AAV9 or AAV2 capsids. Virions were produced by transfecting HEK293 cells with (i) the adenovirus helper plasmid (pXX6-80), (ii) the AAV packaging plasmid encoding the rep2 and the cap2 or the cap9 genes, and (iii) the AAV2 shuttle plasmid containing the gene encoding GFP or mSEAP in a ss or sc genome. Recombinant vectors (rAAV) were purified by double-CsCl ultracentrifugation followed by dialysis against the formulation buffer of the vector stocks, namely phosphate-buffered saline containing 0.5 mM MgCl2 and 1.25 mM KCl (PBS-MK; five buffer changes, 3 hours per round of dialysis). Physical particles were quantified by real-time PCR. Vector titers are expressed as viral genomes per milliliter (vg/ml).

### Peripheral Administration of AAV Vectors

In adults, AAV vectors were administered peripherally by injection into the tail vein at 8 weeks of age. The animals were restrained in a tube, facilitating manipulation of the tail. A 30-gauge needle attached to a 1 ml syringe was inserted into the tail vein and 500 µl of the viral solution was injected over a period of about 30 seconds.

### Real-time PCR Quantification of Vector Genome Copy Number in the Retina

Enucleated whole eyes were snap-frozen in liquid nitrogen and stored at −80°C until further processing. Frozen tissues were lysed in 700 µl of nuclear lysis buffer (Wizard genomic DNA extraction kit, Promega, Charbonnières-les-Bains, France). Tissues were homogenized by four 30-second pulses with an Ultra-Turrax homogenizer, to ensure complete lysis. Cell membranes and debris were pelleted by centrifugation for 2 minutes at 10,000×*g* and 4°C. A sample of the supernatant was collected for the mSEAP quantification assay, and genomic DNA containing the AAV vector genome was purified according to the manufacturer’s instructions. For each sample, we used 72 ng of genomic DNA as the template. Vector genome copy number was determined by a real-time PCR assay with primers and a probe corresponding to the inverted terminal repeat region (ITR) of the AAV vector genome common to ss and scAAV vectors. The sequences of the primer pair and the Taqman probe were 5′-CTCCATCACTAGGGGTTCCTTG-3′ (AAV-Fw), 5′-GTAGATAAGTAGCATGGC-3′ (AAV-Rev) and 5′-TAGTTAATGATTAACCCAA-3′ (AAV-MGBprobe). Amplification of the Titin gene was used to normalize the results with respect to the number of AAV vector genome copies per cell. The sequences of the primer pair and the Taqman probe were 5′-AAAACGAGCAGTGACGTGAGC-3′ (Titin-Fw), 5′-TTCAGTCATGCTGCTAGCGC-3′ (Titin-Rev) and 5′-TGCACGGAAGCGTCTCGTCTCAGCT-3′ (Titin-VIC/TAMRAprobe). Serial dilutions of the rAAV vector plasmid were used to generate a standard curve for the determination of vector genome copy numbers. Real-time PCR was carried out and results were analyzed with the ABI Prism 7700 sequence Detection System (Applied Biosystems, Foster City CA, USA).

### mSEAP Quantification Assay

mSEAP activity in the eye lysate supernatant was quantified in a chemiluminescence assay. Endogenous alkaline phosphatase was inactivated by heating at 65°C for 5 minutes and the heat-resistant mSEAP activity was measured by adding reaction buffer and the CPSD chemiluminescence substrate of the Tropix system, according to the manufacturer’s instructions (Applied Biosystems, Foster City CA, USA). Chemiluminescence was then determined with a luminometer (Perkin Elmer). Activity levels are expressed as ng of mSEAP and were determined by comparison with a standard curve for purified human placental alkaline phosphatase. Results were normalized on the basis of protein concentration, determined with the Nano-orange protein quantification assay (Invitrogen, Cergy-Pontoise, France).

### Histological Processing

Mice were deeply anesthetized with 10 mg.kg^−1^ xylazine and 100 mg.kg^−1^ ketamine and transcardially perfused with 0.1 M phosphate-buffered saline (PBS) followed by ice-cold 4% paraformaldehyde in PBS. The eyes were enucleated and post-fixed by overnight incubation in the same fixative, cryoprotected by overnight incubation in 15% sucrose in PBS, frozen in cold isopentane (−50°C) and cut on a cryostat into 14 µm sections, which were stored at −80°C until further processing. For immunofluorescence labeling, sections were rinsed twice in PBS, blocked by incubation for one hour in PBS supplemented with 10% normal goat serum (NGS) and 0.2% Triton X-100 and incubated overnight at 4°C with primary antibodies in PBS supplemented with 1% NGS and 0.2% Triton X-100. Anti-GFP antibody (1∶800; Abcam, Cambridge, UK), anti-Brn-3a antibody (1∶1′000; Millipore, Billericay MA, USA) and anti-Chx10 antibody (1∶300; Santa Cruz Biotechnology, Santa Cruz CA, USA) were used. Sections were then rinsed in PBS, incubated with fluorescently labeled secondary antibodies (Alexa-Fluor conjugates, 1∶1,000; Invitrogen, Cergy-Pontoise, France), labeled with 4′,6′-diamidino-2-phenylindol (DAPI) and mounted in Mowiol 4–88 reagent (Sigma-Aldrich, Lyon, France) and covered with a coverslip for examination under a DM6000-B epifluorescence microscope (Leica Microsystèmes, Nanterre, France). Brn-3a- and GFP-positive cells were counted by eye at 20× magnification.

## Results

We investigated the efficiency of retina cell transduction following the intravenous delivery of scAAV9 vectors in adult mice, by injecting a scAAV9 vector expressing the murine secreted alkaline phosphatase (mSEAP) under the transcriptional control of the cytomegalovirus (CMV) promoter (10^12^ vector genomes [vg] per mouse) into the tail vein of eight-week-old mice. Four weeks after injection, quantitative PCR analysis on genomic DNA from the whole eyes showed that the vector was present in eye tissue (0.320+/−0.184, n = 3). Furthermore, significant mSEAP activity was detected in eye protein extracts from the scAAV9-treated mice (10.15+/−5.86 pg of mSEAP per mg of protein, n = 3). These results strongly suggest that scAAV9 was able to reach the ocular tissues after intravenous injection in adult mice, probably by crossing mature blood-eye barriers.

**Figure 1:**
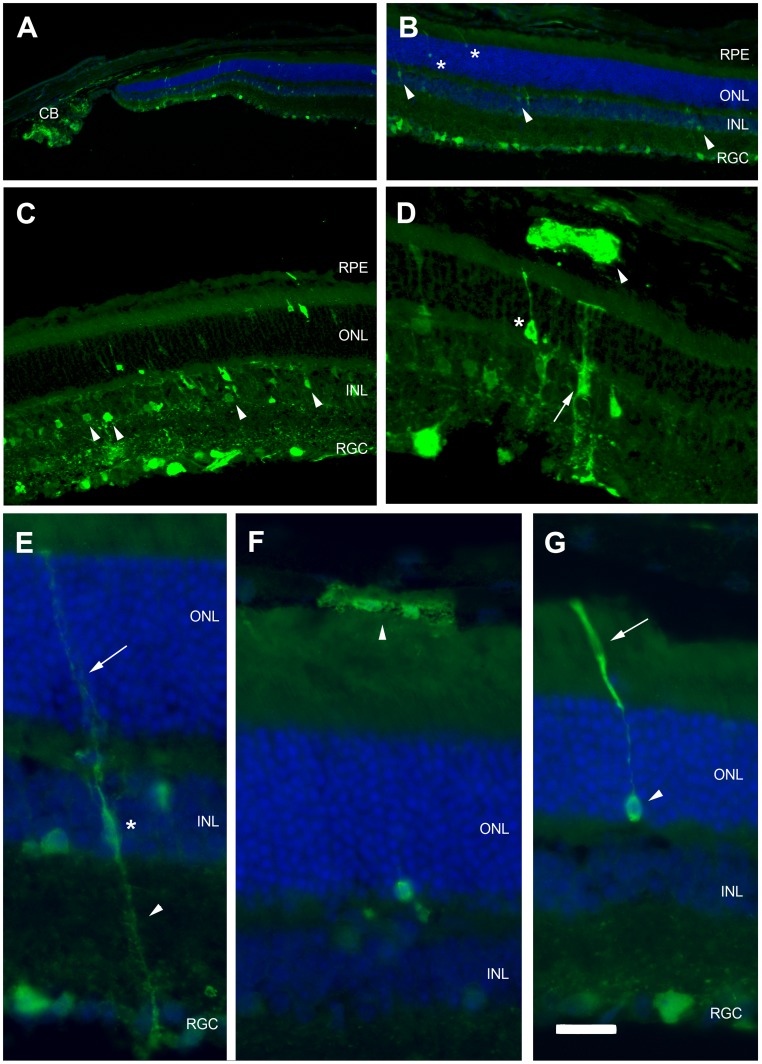
GFP expression in the retina after the intravenous delivery of scAAV9-GFP in adult mice. Retinal cross sections were treated for GFP immunofluorescence (green) and counterstained with DAPI (blue), four weeks after the injection of 2×10^12^ vg of scAAV9-GFP into the tail veins of eight-week-old mice. GFP was detected in all retina layers (A–D) and in the ciliary bodies (CB in A). Transduction efficiency was particularly high in the RGC layer (B–C) but GFP was also expressed in the various cell types of the inner nuclear layer (INL), including cells with the morphology of bipolar cells (arrowheads in C) and of Müller cells (arrows in D). Rare GFP-positive photoreceptors (asterisks in B and D) and RPE cells (arrowheads in D and F) were also detected. (E–G) High magnification of GFP-positive (E) Müller cells, (F) RPE cells and (G) photoreceptors. RPE: retinal pigment epithelium; ONL: outer nuclear layer; INL: inner nuclear layer; RGC: retinal ganglion cell layer. Scale bar: 200 µm in A and B; 70 µm in C; 50 µm in D; 20 µm in E–G. A, B and E–G are epifluorescence images; C and D are confocal images.

**Figure 2:**
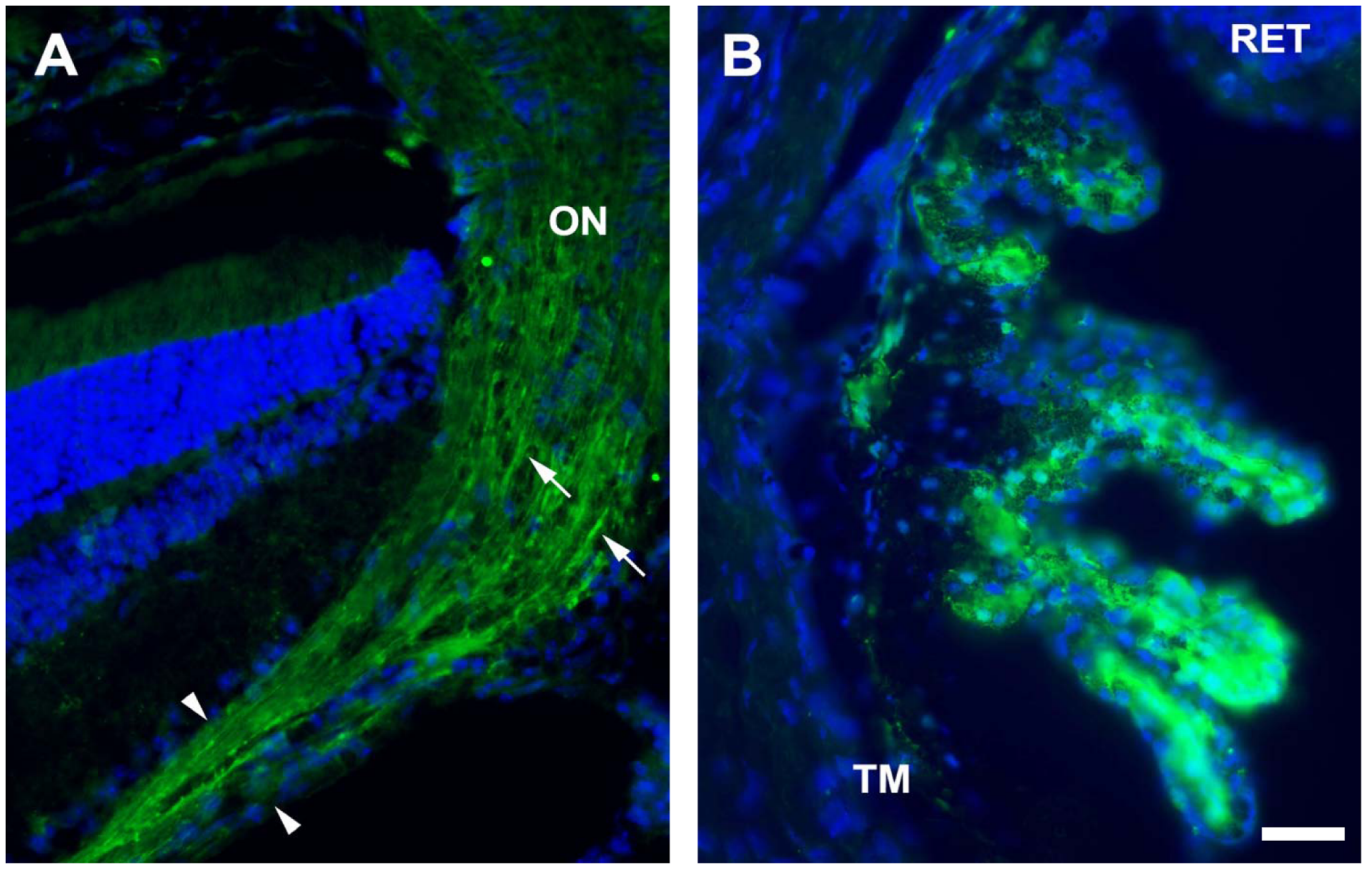
GFP expression in the optic nerve and the ciliary body of intravenous scAAV9-GFP injected adult mice. Representative cross sections of the optic nerve (A) and the ciliary body (B) treated for GFP immunofluorescence (green) and stained with DAPI (blue), four weeks after the injection of 2×10^12^ vg of scAAV9-GFP into the tail veins of eight-week-old mice. In (A), the boundaries of the retinal nerve fiber layer (originating from the RGC) are clearly demarcated by their pattern of GFP expression (arrowheads) (arrows: GFP-positive axons in the optic nerve). (B) High magnification of the ciliary body, showing strong GFP expression in the epithelial cells. ON: optic nerve; RET: retina; TM: trabecular meshwork. Scale bar: 100 µm.

We tested this hypothesis by analyzing the pattern of retinal cell transduction after the intravenous injection of a scAAV9 vector expressing the green fluorescent protein (GFP) under transcriptional control of the CMV promoter into adult mice. We injected scAAV9-CMV-GFP (2×10^12^ vg per mouse) into the tail vein of eight-week-old mice (n = 6) and assessed transgene expression in immunofluorescence analyses carried out four weeks later. Multiple layers of the retina expressed GFP to various extents in all the animals. GFP was detected in the choroid, the RPE, the photoreceptor and outer nuclear layers (ONL) (rod/cones), the outer plexiform and inner nuclear layers (INL) (bipolar and horizontal cells), the inner plexiform and RGC layers (amacrine and ganglion cells), for example (Figure 1). Strong GFP expression was also detected along the ganglion cell axons forming the optic nerve and within the ciliary bodies (Figure 2). No specific immunostaining was detected in control mice that received no injection (data not shown).

**Figure 3:**
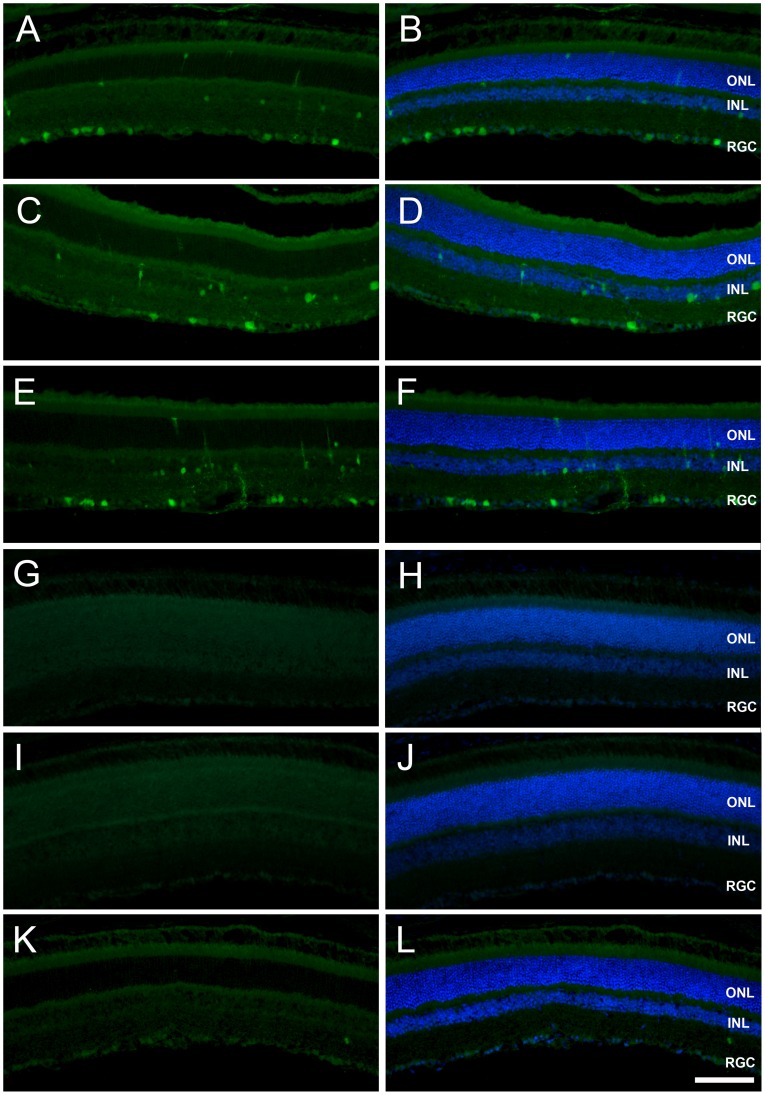
Systemic injection of AAV serotype 2 does not lead to transduction of the neural retina. GFP expression in representative cross-sections of the retina of adult mice one month after systemic administration of 2.10^12^ vg scAAV-GFP of serotype 9 (A–F) or serotype 2 (G–L) in adult mice (n = 3 per condition). GFP expression was detected in the neural retina in all mice from the serotype 9 treated-group (panel A to F are from three different animals). As expected, the highest transduction efficiency was observed at the level of the RGC layer. In contrast, no GFP expression was detected in the retina after AAV serotype 2 injection (panels G to L are from three different animals). Left panels: GFP immunofluorescence; right panels: merged view of GFP (green) and dapi (blue). ONL: outer nuclear layer; INL: inner nuclear layer; RGC: retinal ganglion cell layer. Scale bar: 100 µm.

To demonstrate the specificity of scAAV9 to transduce retinal neurons after peripheral administration in adult mice, another serotype was administered according to the same experimental design. We selected AAV serotype 2, a vector which does not cross the BRB and which is particularly efficient for ganglion cell transduction following intravitreal injection in mice [33]. New batches of AAV2 and AAV9 vectors were produced and injected into the tail vein of adult mice (2×10^12^ vg/mice, n = 3 mice for each serotype). One month after injection, numerous GFP expressing retinal cells were clearly detected in the 6 eyes of the scAAV9-injected mice (Figure 3). In accordance to the previous experiment, the retinal cell layer most efficiently transduced by scAAV9 was the RGC layer (Figure 3). In contrast, no GFP expression was detected in the retina of any of the 6 eyes from the scAAV2-injected mice, which confirmed that the BRB was not disrupted by the gene transfer procedure.

**Figure 4:**
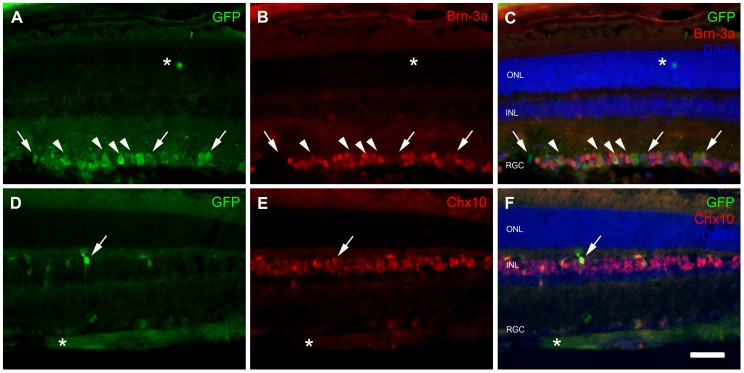
GFP expression in RGC and bipolar cells. Representative retina sections from adult mice treated for double-immunofluorescence analysis four weeks after the injection of 2×10^12^ vg of scAAV9-GFP vectors into the tail vein. (A–C) Double-labeling of transduced RGCs for GFP (green) and Brn-3a (red) (arrowheads indicate double-labeled RGCs and arrows indicate cells expressing GFP only). A transduced photoreceptor is highlighted with an asterisk. (D–F) Double-labeling of bipolar cells for GFP (green) and Chx10 (red). The arrow indicates a Chx10-positive bipolar cell with high levels of GFP. The retinal nerve fiber layer (RNFL), labeled for GFP due to the transduction of upstream RGC, is indicated by an asterisk. No RGC can be seen on this panel which illustrates the central part of the retina (as demonstrated by the thickness of the RNFL). ONL: outer nuclear layer; INL: inner nuclear layer; RGC: retinal ganglion cell layer. Scale bar: 50 µm.

We then investigated the nature of the GFP-positive cells in the RGC layer, by double-labeling retina sections with antibodies directed against GFP and Brn-3a, a POU Domain transcription factor specifically expressed in the nuclei of the retinal ganglion cells within the retina [34]. A large proportion of the GFP-positive cells located in the RGC layer were found to be retinal ganglion cells (Figure 4 A–C). We also investigated the colocalization of GFP and Chx10, a transcription factor specifically expressed in the nucleus of bipolar cells [35]. This analysis identified the GFP-positive cells located in the INL as bipolar cells (Figure 4 D–F). It is noteworthy that only a negligible number of ganglion cell were visible on the latter sections taken from the central part of the retina (as denoted by the thickness of the retinal nerve fiber layer [RNFL]). At this level of the retina the number of transduced-RGC is low and not representative of the whole retina.

We quantified the efficiency of gene transfer to the retinal ganglion cells of the RGC layer (the most efficiently transduced retina layer), by determining the number of GFP-positive cells, the number of Brn-3a-positive cells, and the number of cells expressing both markers, on transverse sections of the retina at the level of the optic nerve (n = 6, one eye per mouse). We found that 222±20 cells per retinal section expressed GFP in the RGC layer, and that 122±9 of these cells also expressed Brn-3a. The Brn-3a-positive cell population was estimated at 271±14 cells per retinal section. Thus, almost 45% of the Brn-3a-positive retinal ganglion cells in the RGC layer were efficiently transduced after the intravenous delivery of scAAV9-GFP in adult mice.

## Discussion

Recombinant scAAV9 is currently considered to be one of the most promising vectors for human gene therapy because this serotype transduces the cells of various organs, including the heart, liver, skeletal muscle, brain and spinal cord, highly efficiently after its systemic administration [24], [25], [36]–[38]. The demonstration of broad gene delivery to neurons after systemic scAAV9 injection [24], [25] and the therapeutic proof-of-principle of this method in a mouse model of SMA [27]–[29] have paved the way for the clinical development of intravenous scAAV9 gene therapy for SMA in Europe and the USA. This study provides the first demonstration that scAAV9 can transduce ocular tissues following its intravenous injection in adult mice. One month after the injection of a scAAV9 encoding a reporter gene in eight-week-old mice, transgene expression was detected in multiple layers of the retina, in the optic nerve and in the ciliary bodies. These findings suggest that scAAV9 may cross the mature blood-eye barrier, which, in adult mammalian eyes, consists of tissue layers separating the neural retina and the transparent refractive media from the circulating blood. Like the BBB, there are two main barrier systems in the eye: one essentially regulating inward movements from the blood into the eye at the level of the ciliary body (the blood-aqueous barrier), and the other preventing outward movement from the retina into the blood (the blood-retinal barrier) [23]. We found that retinal ganglion cells were the principal cells transduced in the retina after the intravenous injection of scAAV9 in adult mice. These findings suggest that scAAV9 may be delivered to the neural retina either directly from the retinal circulation, by crossing the blood-retinal barrier, or indirectly, entering the aqueous and vitreous humors via the ciliary bodies –the structural equivalent of the blood-aqueous barrier– to reach its final destination, the retinal cells. The ciliary processes and the adjacent retinal cells appeared to be strongly transduced after intravenous scAAV9 injection, suggesting that at least some of the vector passed across the tight junctions between the non pigmented cells of the ciliary epithelium.

These findings are of particular importance because systemic AAV9-mediated transduction of the retina has previously been reported to be dependent on the age of the animal, with efficient transduction observed only in neonatal or fetal animals [39]–[42].

Such discrepancies between our data and previous work from several groups may be due to the use in our study of a self-complementary genome-based AAV9, or to species- differences in the vector tropism. For example, Bostick *et al*. showed that the systemic injection of single-stranded (ss) AAV9 mediated gene transfer to the inner layer of the retina in neonatal mice, but that systemic ssAAV9 gene transfer was inefficient in adults [39], suggesting the superiority of the scAAV9 *versus* its single-stranded counterpart. Also, RPE cells but not other retinal cells were found to be targeted when scAAV9 vectors were delivered to other species such as the cat or the dog [40]. The use of different promoters could also explain the disparity between the studies. In the present study, we used a CMV promoter, which is known to drive high and constitutive expression of foreign genes in a variety of neurons and in retinal ganglion cells in particular [43], while for instance Bostick et *al*. used a RSV promoter in adults and neonates [39], and Dalkara et *al*. used a CAG promoter in neonates [42]. The method of AAV preparation (CsCl or Iodixanol purification) could also impact both the transduction pattern and efficiency by inducing changes in the vector tropism [44]. Finally, the use of different doses of vector could also be the cause of differences between studies, but this remains particularly difficult to establish because of the various titration methods used in different laboratories.

This first report of efficient retinal gene delivery by systemic AAV9 treatment in adult animals might have important implications for clinical applications. Indeed, the possibility of targeting retina cells in adult patients soon after the onset of symptoms is an essential prerequisite of gene therapy for most retinal diseases. In this context, systemic AAV9 gene delivery may be indicated, in particular, for the treatment of Leber’s hereditary optic neuropathy (LHON), a genetic disease of young adults leading to the preferential degeneration of RGCs and their axons (optic nerve).

Another advantage of systemic gene therapy is its potential application to retinal diseases for which intravitreal or subretinal injections would be too risky. For example, high intraocular pressure (IOP) in glaucoma is a major risk factor thought to play a major role in RGC degeneration. Local gene vector injection, which would further increase IOP and induce an inflammatory reaction, could thus be replaced by the systemic delivery of scAAV9 vectors. This route of administration would also be particularly advantageous for the two forms of age-related macular degeneration (AMD), in which abnormal neovascularization of the choroid is associated with a risk of bleeding and irreversible damage to the retina (“wet” AMD) or the accumulation of cellular debris (drusen) between the retina and the choroid (“dry” AMD). Both forms of AMD lead to retinal detachment and could benefit advantageously from a systemic gene therapy.

In the present study, the use of a ubiquitous promoter resulted in a non-specific expression of the transgene in the whole retina and in a number of nervous and non-nervous organs [24] following scAAV9 systemic delivery. The use of this method for specific delivery of transgenes to the retina might therefore involve utilization of cell-specific promoters, such as the Thy1.1 promoter for RGC specific expression [45], or the myocilin promoter, which may allow preferential expression in the ciliary body [46]. Although tissue specific promoters generally drive lower expression than ubiquitous promoters in many tissues, in some cases, tissue-specific promoters were found to be superior to ubiquitous promoters in vitro and in vivo [47], [48]. In the retina, Miyoshi et al. (1997) found the rhodopsin promoter to be significantly superior to CMV in vivo to drive transgene expression in the photoreceptors (x2.5 higher levels of expression in the photoreceptors than the CMV promoter) [49].

However, in addition to its possible use to treat diseases limited to the eye, systemic injection of scAAV9 vectors might also be useful for treating pathological conditions affecting both the eye and other organs. For example, systemic gene therapy may be a particularly suitable approach to the treatment of syndromic retinitis pigmentosa (Usher syndrome), a disease in which both the retina and the sensory neurons of the ear are impaired [50], and for the treatment of many lysosomal storage diseases, such as Gaucher’s disease, which affects peripheral organs (the lung, liver and spleen in particular) and in which patients also present neurological symptoms and eye problems, such as glaucoma. The Bardet-Biedl syndrome, a genetic multisystem disorder, would also benefit in an advantageous way from systemic gene therapy using AAV9 vectors [51].

Another important finding of this study was the highly efficient transduction of the ciliary body, an epithelium within the eye that is responsible for producing the aqueous humor. Following systemic scAAV9 transduction, ciliary bodies could thus be used as biological pumps, to ensure the long-term secretion of therapeutic proteins (such as anti-VEGF antibodies [52]) into the anterior and posterior chambers of the eye or into the vitreous humor [53].

In summary, we report here the capacity of intravenous scAAV9 to target multiple retinal layers in adult animals in a non invasive manner, with a predominant transduction of the retinal ganglion cells, the optic nerve and the ciliary bodies. Improving the efficiency of transduction in all retinal layers (particularly the photoreceptors), for example by the use of tyrosine to phenylalanine mutant AAV9 [41], and inducing specificity of expression to the retina using cell-specific promoters would present this intravascular approach as a basis for alternative developments to current subretinal or intravitreal injection. Attempts to translate this strategy into clinical practice for humans should also take into account possible species differences in the retinal tropism of AAV9 vectors.
